# Targeting lncRNA MALAT1: A Promising Approach to Overcome Metabolic Syndrome

**DOI:** 10.1155/2024/1821252

**Published:** 2024-10-28

**Authors:** Gulandanmu Aihemaiti, Ning Song, Junyi Luo, Fen Liu, Jianaerguli Toyizibai, Niyaziaili Adili, Chang Liu, Wei Ji, Yi-Ning Yang, Xiaomei Li

**Affiliations:** ^1^Department of Cardiology, The First Affiliated Hospital of Xinjiang Medical University, Urumqi, China; ^2^Xinjiang Key Laboratory of Cardiovascular Disease Research, Clinical Medical Research Institute of First Affiliated Hospital of Xinjiang Medical University, Urumqi, China; ^3^People's Hospital of Xinjiang Uygur Autonomous Region, Urumqi, China

**Keywords:** dyslipidemia, dysregulated glucose homeostasis, hypertension, insulin resistance, LncRNA MALAT1, metabolic syndrome

## Abstract

Metabolic syndrome (MetS) is a collection of metabolic abnormalities including insulin resistance, atherogenic dyslipidemia, central obesity, and hypertension. Recently, long noncoding RNAs (lncRNAs) have emerged as pivotal regulators of metabolic balance, influencing the genes associated with MetS. Although the prevalence of insulin resistance is rising, leading to an increased risk of type 2 diabetes mellitus (T2DM) and its vascular complications, there is still a notable gap in understanding the role of lncRNAs in the context of clinical diabetes. Among lncRNAs, lung adenocarcinoma metastasis-associated transcript 1 (MALAT1) has been identified as a significant regulator of metabolism-related disorders, including T2DM and cardiovascular disease (CVD). This review explores the mechanism of lncRNA MALAT1 and suggests that targeting it could offer a promising strategy to combat MetS, thereby enhancing the prognosis of MetS.

## 1. Introduction

MetS, or metabolic syndrome, is rapidly becoming a global health concern owing to its escalating prevalence [1, 2]. Historically, Reaven first introduced the concept of ‘syndrome X,' which he later renamed MetS. He postulated that this syndrome plays a pivotal role in the development of type 2 diabetes mellitus (T2DM) and cardiovascular disease (CVD), primarily due to target tissue resistance to insulin action [3]. MetS is essentially a collection of clinical conditions, including abdominal obesity, insulin resistance (IR), T2DM, hypertension, and atherogenic dyslipidemia [4]. Among these, abdominal obesity and IR are increasingly recognized as central features of the syndrome [5]. This syndrome is associated with accelerated atherosclerosis, which results from chronic inflammation and vascular endothelial dysfunction, leading to significantly increased cardiovascular risk [4]. Lipid disorders, which often manifest as abnormalities in serum cholesterol or triglyceride levels, are frequently observed in clinical settings and have significant influences in MetS [6]. Interestingly, hypertension is also influenced by a combination of genetic and environmental factors which include high-fat diet. The high prevalence of hypertension in MetS suggests a complex interplay between dyslipidemia and diabetes. These conditions often exhibit shared abnormalities in inflammation and oxidative stress, potentially explaining their interconnectedness as cardiac risk factors [7]. Consequently, as the incidence of this spectrum of diseases continues to increase, it significantly contributes to global morbidity and imposes a substantial socioeconomic burden.

Current therapeutic approaches for MetS focus on lifestyle improvements, medications, and surgical interventions for complications. However, the persistence of unhealthy lifestyles, coupled with a lack of specific biomarkers, has contributed to a rising incidence rate. It is imperative for clinicians to deepen their understanding of the molecular mechanisms underlying MetS to develop effective treatment strategies. Long noncoding RNAs (lncRNAs) have emerged as pivotal regulators of various biological processes, including apoptosis, glycolipid metabolism, and oxidative phosphorylation. Given the multifaceted roles of lncRNAs, it is crucial to study their physiological functions in specific tissues across all organisms.

Metastasis-associated transcript 1 (MALAT1) may be one of the key lncRNAs regulated by MetS. Numerous previous studies on MALAT1 have focused on the mechanistic area of its development in different tumors. A comprehensive review and meta-analysis revealed that MALAT1 overexpression is linked to poor outcomes in nonsmall cell lung carcinoma (NSCLC), suggesting its potential as a prognostic marker for NSCLC [8]. MALAT1 has also been implicated in enhancing angiogenesis in thyroid cancer by modulating tumor-associated macrophage fibroblast growth factor 2 (FGF2) [9]. In addition, MALAT1 has been found to be associated with tumor invasion and distant metastasis, which may be accomplished through interactions with different signaling pathways [10, 11]. Zhang et al. found that MELLT3 regulated MALAT1 may enhance proliferation and metastasis in osteosarcoma cells [12]. Interestingly, MiR-423-5p could prevent MALAT1-regulated metastasis through a ceRNA network [8]. In addition, MALAT1 has also been found to regulate various signaling pathways, including NF-KB, Wnt/*β*-catenin, Notch, and PI3K/AKT/mTOR, contributing to cancer development [11].

Recently, the crosstalk between MALAT1 and MetS was identified. In cardiovascular health, lncRNA MALAT1 has been correlated with factors such as the Gensini score and cholesterol levels in patients [13]. It also plays potential roles in obesity by regulating lipogenic and adipogenic genes [14] and has been linked to cerebrovascular pathologies in ischemic stroke [9]. Furthermore, MALAT1 has been implicated in lipid accumulation in HepG2 cells [15] and in the regulation of cellular survival, proliferation, and migration in pancreatic carcinomas [16].

**Figure 1:**
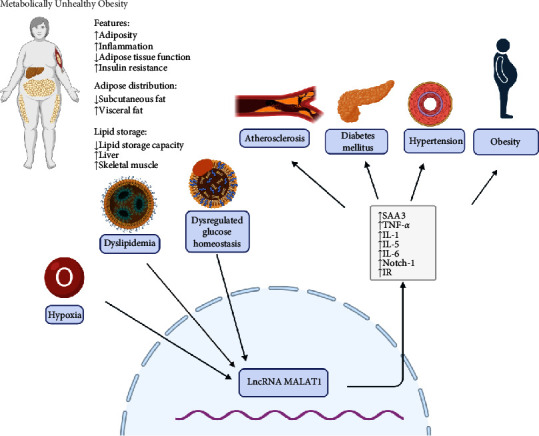
Characteristics of MetS and the relationship between lncRNA MALAT1.

In summary, current research suggests that MALAT1 may influence MetS through gene regulation, inflammation, and apoptosis, indicating that our understanding of MALAT1 is still evolving. This review aims to discuss the role of lncRNA MALAT1 in MetSs and their associated conditions, emphasizing its potential as a novel biomarker and therapeutic target for these diseases (Figure 1).

## 2. Biogenesis of LncRNA MALAT1

MALAT1, also referred to as NEAT2 (nuclear-enriched abundant transcript 2), is a long (∼8 kb) ncRNA predominantly located in the nucleus, specifically within nuclear speckles. Initially identified in nonsmall cell lung carcinoma, MALAT1 orthologs were recently found in zebrafish [12]. Its maturation and stabilization are based on the accurate processing of RNase P, which simultaneously initiates the biosynthesis of 3′ cytoplasmic MALAT1-associated small cytoplasmic RNAs (mascRNAs) [17]. This lncRNA has previously been associated with various malignancies, including lung, gastric, and thyroid cancer. Interestingly, increased evidence suggests that MALAT1 also plays a crucial role in the onset and pathological development of MetS. We will describe the potential role and future expectations of MALAT1 from different MetS types.

## 3. Function and Role of MALAT1

MALAT1 dysregulation affects various cellular processes, leading to abnormal cell proliferation and migration characteristics in carcinomas. Recent studies have also highlighted MALAT1's role in obesity, dysregulated glucose homeostasis, dyslipidemi, and hypertension, collectively termed MetS. The following sections describe how MALAT1 interacts with dysregulation of metabolism and function to summary comprehensive mechanisms and bring about new insights.

### 3.1. MALAT1 and MetS: Focus on Obesity

Endothelial dysfunction, which is prevalent in obese children and adolescents, increases the risk of CVD. Exercise, known for its protective effects against endothelial dysfunction, achieves this in part by modulating specific noncoding RNAs. A study suggests that the MALAT1/miR-320a axis plays a role in the beneficial effects of exercise on endothelial function in this demographic [18].

Furthermore, the MALAT1 rs3200401 single nucleotide polymorphism (SNP) has been identified as a risk factor for childhood obesity, particularly in dominant and allelic models. Notably, the CT heterozygous genotype was correlated with increased BMI and IR [19].

Additionally, research has indicated that MALAT1, when induced by saturated fatty acids (SFAs), can exacerbate myocardial inflammatory injury. However, its downregulation can mitigate this injury through the miR-26a/HMGB1/TLR4/NF-*κ*B axis, shedding light on the mechanisms underlying myocardial lipotoxic injury [20].

### 3.2. MALAT1 and Dysregulated Glucose Homeostasis

Dysregulation of glucose metabolism has been linked to numerous human diseases, including cancer and diabetes. Diabetes, a prevalent metabolic disorder, can lead to long-term hyperglycemia and subsequent microvascular lesions [21]. Notably, hyperglycemia in MetS is not limited to those diagnosed with diabetes. It also includes individuals with fasting blood glucose levels ≥ 6.1 mmol/L, so they are emerging as pivotal regulators of various biological processes, influencing mRNA translation, epigenetic modifications, and transcription factor sequestration. Recent studies have highlighted their role in metabolic homeostasis [22]. Diabetic hearts exhibit metabolic changes such as intramyocardial triglyceride accumulation and increased lipid oxidation. They also show reduced glucose utilization and IR [23, 24].

Under diabetic conditions, MALAT1 expression is elevated in cardiomyocytes and myocardial tissues [24]. Silencing MALAT1 alleviates cardiomyocyte damage by targeting NLRP3 (NOD-like receptor protein 3). Moreover, MALAT1 can amplify oxidative stress in PC12 cells, exacerbating cell damage caused by chemical hypoxia [25].

In studies involving human umbilical vein endothelial cells (HUVECs) exposed to varying glucose levels, an increase in MALAT1 expression was observed under high glucose conditions. This increase was associated with elevated levels of inflammatory mediators, suggesting MALAT1's role in glucose-induced inflammation [26]. Another study linked elevated MALAT1 levels with factors such as weight loss, smoking, and alcoholism in T2DM patients, suggesting its potential impact on disease severity [27].

Maintenance of blood glucose levels largely depends on insulin regulation. Insulin, a hormone secreted by pancreatic *β*-cells, is crucial for systemic metabolism and is implicated in various metabolic diseases [28]. Dysregulation of insulin signaling disrupts glucose homeostasis. Studies have shown that MALAT1 plays a role in IR and affects target organs [29]. For instance, exercise has been found to modulate MALAT1 levels, influencing resistin and thereby affecting IR [30]. However, the exact mechanisms by which MALAT1 regulates IR remain to be fully elucidated and warrant further investigation (Figure 1).

### 3.3. MALAT1 and Dyslipidemia

Dyslipidemia, characterized by elevated triglyceride levels, increased low-density lipoprotein (LDL), and decreased high-density lipoprotein (HDL) levels, is a significant risk factor for coronary artery disease, stroke, and MetS. Long-term epidemiological studies have consistently shown that individuals with healthier lifestyles and favorable lipid profiles have a reduced incidence of coronary heart disease. Proper management and prevention of dyslipidemia can significantly alter cardiovascular outcomes [31].

Excessive and abnormal fat accumulation is associated with adverse health outcomes, including an increased risk of severe conditions such as T2DM. Both low HDL cholesterol and hypertriglyceridemia are implicated in the development and progression of diabetic kidney disease [32].

A case-control study by Kazemi et al. [33] on individuals aged ≤ 50 years with acute myocardial infarction found a higher prevalence of MetS in the case group than in the control group, with high triglyceride levels being the most common component. Another study by Ramesh et al. [34] identified a reduction in HDL levels as the most prevalent factor in MetS among patients with acute myocardial infarction.

Furthermore, research has shown that MALAT1 expression increases in exosomes from oxidized LDL (ox-LDL)-treated human HUVECs. When these exosomes are cocultured with neutrophils, they enhance the formation of neutrophil extracellular traps (NETs), a process modulated by exosomal MALAT1.

Moreover, exosomes derived from ox-LDL-treated HUVECs induced an inflammatory response, hyperlipidemia, and NETs release in a rat atherosclerosis model. In essence, exosomal MALAT1 from ox-LDL-treated endothelial cells triggers NETs formation, thereby exacerbating atherosclerosis [32].

### 3.4. MALAT1 and Hypertension

Hypertension is an escalating global health concern [35]. Its pathophysiology is multifaceted, involving factors such as overactivation of the renin-angiotensin-aldosterone system, inflammation, and oxidative stress, all of which play pivotal roles. Recent studies have highlighted the role of MALAT1 in hypertension, particularly in vascular lesions and remodeling in hypertensive mice. Notably, MALAT1 expression was elevated in hypertensive rats, suggesting its potential as a diagnostic marker for hypertension.

Inhibition of Notch-1 and downregulation of MALAT1 resulted in a decrease in the expression of elements related to endothelial function, inflammation, and oxidative stress. This suppression also prevented apoptosis of aortic endothelial cells in hypertensive mice. These findings suggest that downregulation of MALAT1 can mitigate vascular damage and remodeling in hypertensive mice, potentially through the inhibition of the Notch signaling pathway [36].

Moreover, inflammation is recognized as a risk factor for hypertension [37]. Recent research has shown that reducing MALAT1 levels can decrease the concentrations of proinflammatory cytokines such as TNF-*α*, IL-6, and IL-1*β*, which are closely associated with hypertension.

**Figure 2:**
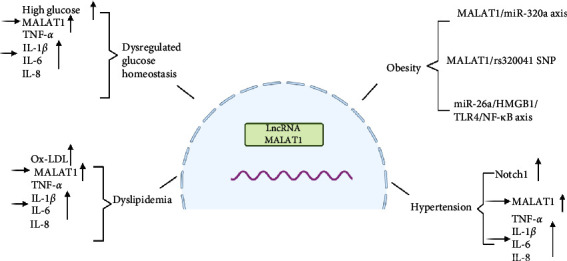
Relevant role of lncRNA MALAT1 in metabolic syndrome.

Further studies on the role of MALAT1 in the proliferation and apoptosis of HUVECs have provided insights into its potential therapeutic implications in hypertension. These studies revealed elevated levels of the lncRNA MALAT1 in the plasma of hypertensive patients [38] (Figure 2).

### 3.5. MALAT1 and Signaling Pathways

**Figure 3:**
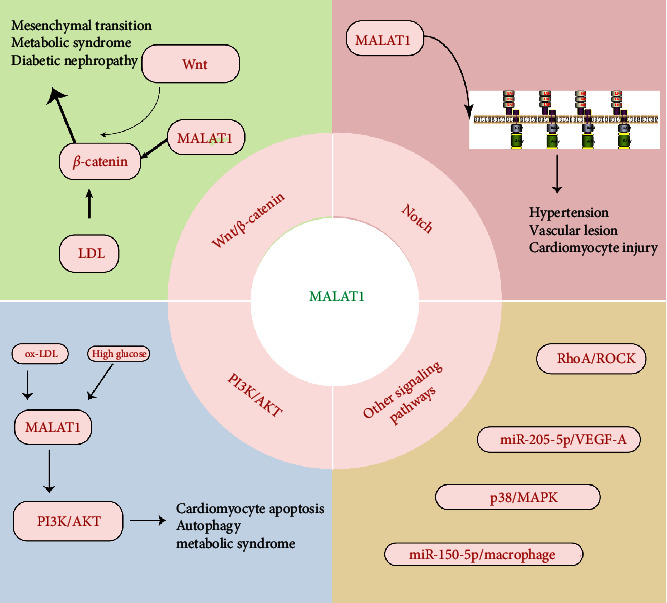
MALAT1 and related classic signaling pathways.

In the progression of MetS, various signaling pathways including Wnt/*β*-catenin, Notch, and PI3K/AKT/mTOR signals play significant roles. In addition, MALAT1 also exert crosstalk with these critical signaling pathways. The following part elucidates the interoperability of MALAT1 and these signaling pathways (Figure 3).

### 3.6. MALAT1 and Wnt/*β*-Catenin-Signaling Pathway

Wnt/*β*-catenin is a signaling pathway that is highly correlated with cell invasion and migration and often plays an important role in epithelial mesenchymal transition. Recent studies have shown its relevance to cardiovascular cell migration and metabolic syndrome. Li et al. found that MALAT1 expression was elevated in myocardial tissues of mice fed a high-fat diet and promoted LDL-induced *β*-catenin nuclear translocation and activation of endothelial mesenchymal transition, which ultimately contributed to the progression of metabolic syndrome and atherosclerosis [39]. MALAT1 was also found to promote myocardial injury through the miR-374a/Sp1/Wnt/*β*-catenin pathway [40]. Zhang et al. indicated that upregulated MALAT1 induced EMT in high glucose-treated HK-2 cells through activating the Wnt/*β*-catenin pathway, finally contributed the pathogenesis of diabetic nephropathy [41]. Interestingly, some herbal medicines have also been found to mitigate the progression of metabolic syndromes such as diabetes by modulating MALAT1 and related pathways. QiHuangYiShen granules may inhibit the MALAT1 expression and Wnt/*β*-catenin pathway, contributed to the alleviation of renal fibrosis and attenuated the podocyte EMT in diabetic nephropathy [42].

### 3.7. MALAT1 and Notch-Signaling Pathway

The Notch pathway often plays a key role in the progression of different tumors, and recent studies have shown that notch also plays an important role in the pathological mechanisms of metabolism-related diseases. Xue et al. indicated that MALAT1 expression was increased in hypertension patients, and decreased MALAT1 may activate the notch pathway and alleviate the vascular lesion and remodeling [36]. The potential link between MALAT1 and hypoxia has also been reported numerous studies, which have found that hypoxic conditions enhance the expression of MALAT1. MALAT1 also exhibited significant crosstalk with notch and PI3K/AKT pathways, contributing to the hypoxia-induced cardiomyocyte injury [43].

### 3.8. MALAT1 and PI3K/AKT-Signaling Pathway

Many literatures report the potential link between MALAT1 and PI3K/AKT and the potential role in metabolism-related diseases. Li et al. found that MALAT1 could induce autophagy in HUVECs with ox-LDL stimulation [44]. Inhibition of MALAT1 can regulate cardiomyocyte apoptosis via PI3K/Akt and alleviates myocardial ischemia-reperfusion [45]. In a high glucose-induced environment, MALAT1 promotes the proliferation and migration functions of cellular vascular endothelial cells via the PI3K/AKT pathway [46]. Interestingly, in type 2 diabetes mices, increased MALAT1 could upregulated the expression of PI3K/AKT, INSR, and IRS-1, thereby alleviating cognitive impairment [47]. These studies demonstrate a link between MALAT1 and metabolic syndrome, the key crosstalk being the PI3K/AKT-signaling pathway.

### 3.9. MALAT1 and Other Signaling Pathways

Many other signaling pathways have also shown links to MALAT1 and the metabolic syndrome. Wang et al. reported the potential crosstalk between MALAT1 and cardiomyocyte apoptosis. MALAT1 promoted the RhoA/ROCK pathway in cardiomyocytes in high glucose stimulation. Knockdown of MALAT1 alleviated oxidative stress and mitochondrial dysfunction, contributing to decreased cardiomyocyte apoptosis [48]. Macrophage-derived exosomal MALAT1 also exhibited crosstalk with diabetes-related complications. High glucose stimulation upregulates the expression level of MALAT1 and inhibits miR-150-5p expression in macrophages, counteracts the inhibitory effect of miR-150-5p on macrophage resistin expression, and promotes pathological progression of diabetes-related vascular disease [49]. Tan et al. elucidate that decrease of MALAT1 improves high-glucose-induced angiogenesis in diabetic retinopathy through miR-205-5p/VEGF-A-signaling pathways. In addition, MALAT1 was also reported to be involved in diabetes-associated intervertebral disc degeneration, which is a diabetes-associated complication. The p38/MAPK-signaling pathway may be a key bridge between MALAT1 and diabetes-associated intervertebral disc degeneration [50].

## 4. Development Prospects

As research in genetic engineering and molecular biology has deepened, lncRNAs have emerged as a promising avenue of exploration. Human diseases often involve intricate networks of signaling pathways and interactions. lncRNAs play a pivotal role in modulating these interactions by connecting various signaling cascades with their diverse interactomes, including proteins, lipids, and RNAs.

While lifestyle modifications, especially dietary changes, remain the primary strategies for managing and treating MetS, a universally effective dietary model is yet to be established [51]. Notably, individuals diagnosed with MetS tend to be younger [52]. A significant challenge in MetS management is the lack of effective biomarkers, which leads to misdiagnosis and suboptimal treatment outcomes [53].

The process of diagnosing MetS is intricate, with diagnostic criteria varying globally. Efforts are ongoing to identify biomarkers for MetS that can be assessed using a single blood sample [54].

To date, only a few researchers have explored the potential of lncRNAs as biomarkers for treating MetS at the gene level. However, a meta-analysis, drawing from both literature and the Gene Expression Omnibus (GEO) database, suggests that the lncRNA MALAT1 could serve as a novel biomarker, not only for various cancers [55] but also for MetS-related diseases.

## 5. Conclusion

Our review highlights the potential of the lncRNA MALAT1 as a promising biomarker and therapeutic target for the diagnosis and treatment of MetS. Given its pivotal role in the pathogenesis of MetS and associated complications affecting vital organs like the heart, understanding the underlying mechanisms of MALAT1 is crucial. Rigorous, in-depth research is essential to fully realize the potential of MALAT1 as both a novel metabolic biomarker and an effective therapeutic agent.

## Data Availability

No data were used to support the findings of this study.
